# Supplementary material for: Spatial Variation in Stream-Water Bacterial Communities Across Sites Differing in Wild Chinese Giant Salamander Detection Frequency
Source: Biology (Basel). 2026 Sep 18;15(18):1647. doi: 10.3390/biology15181647 (PMC13604818; doi:10.3390/biology15181647)
Supplement: Supplementary file 1 [file biology-15-01647-s001.zip › Data Related to “Spatial Variation in Stream-Water Bacterial Communities across Sites Differing in Wild Chinese Giant Salamander Detection Frequency”/Microbial Data/report/src/hyperlinks/QC_methods_EN.pdf]

Method

The method is only used as a reference for publication purpose.

Customers are responsible for the related risks of duplicate checking.

1 Experimental Procedure

1.1 Sample Quality Control

Please refer to QC report for methods of sample quality control.

1.2 Library Construction, Quality Control and Sequencing

Sequencing libraries were generated and indexes were added to attribute sequences to each sample. Briefly, PCR amplification of targeted regions was performed by using specific primers connecting with barcodes. The PCR products with proper size were selected by 2% agarose gel electrophoresis. Same amount of PCR products from each sample was pooled, end-repaired, A-tailed and further ligated with Illumina adapters. Libraries were sequenced on a paired-end Illumina platform to generate paired-end raw reads.

Subsequently, library quality was assessed and quantified by QPCR. Quantified libraries will be pooled and sequenced, according to effective library concentration and data amount required.

2 Bioinformatics Analysis Pipeline

2.1 Paired-end reads assembly and quality control

2.1.1 Data split

Paired-end reads was assigned to samples based on their unique barcode and truncated by cutting off the barcode and primer sequence.

2.1.2 Sequence assembly

Paired-end reads were merged using FLASH (http://ccb.jhu.edu/software/FLASH/) (Magoc T et al.,2011), a very fast and accurate analysis tool, which was designed to merge paired-end reads when at least some of the reads overlap the read generated from the opposite end of the same DNA fragment, and the splicing sequences were called raw tags.

2.1.3 Data Filtration

Quality filtering on the raw tags were performed using the fastp software to

obtain high-quality Clean Tags (Bokulich NA et al.,2012).

2.1.4 Chimera removal

The tags were compared with the reference database(Silva database (16S/18S), https://www.arb-silva.de/; Unite Database(ITS), https://unite.ut.ee/) using UCHIME Algorithm (http://www.drive5.com/usearch/manual/uchime_algo.html)to detect chimera sequences, and then the chimera sequences were removed (Edgar RC et al.,2011). Then the Effective Tags finally obtained.

For customer primer-based projects, chimeric sequences are by default detected and removed by alignment against the Silva annotation database (https://www.arb-silva.de/).

If the customer provides paired-end raw data, chimeras will be removed using the corresponding database based on the specified target region: the Silva database (https://www.arb-silva.de/) for 16S/18S, and the Unite database (https://unite.ut.ee/) for ITS.If single-end data is provided, chimera removal is determined by data quality:no chimera removal will be performed for pre-processed clean data, while chimeras will be removed using the corresponding database based on the target region for unprocessed raw data.

3 Reference

Bokulich NA, Subramanian S, Faith JJ, et al. Quality-filtering vastly improves diversity estimates from Illumina amplicon sequencing.Nature Methods. 2012;10(1):57-59. doi:10.1038/nmeth.2276.

Edgar RC, Haas BJ, Clemente JC, Quince C, Knight R. UCHIME improves sensitivity and speed of chimera detection.Bioinformatics. 2011;27(16):2194-2200. doi:10.1093/bioinformatics/btr381.

Magoc T, Salzberg SL. FLASH: fast length adjustment of short reads to improve genome assemblies.Bioinformatics. 2011;27(21):2957-2963. doi:10.1093/bioinformatics/btr507.
